# Biting rates and relative abundance of *Simulium* flies under different climatic conditions in an onchocerciasis endemic community in Ghana

**DOI:** 10.1186/s13071-020-04102-5

**Published:** 2020-05-06

**Authors:** Kenneth Bentum Otabil, Samuel Fosu Gyasi, Esi Awuah, Daniels Obeng-Ofori, Seth Boateng Tenkorang, Justice Amenyo Kessie, Henk D. F. H. Schallig

**Affiliations:** 1 Department of Basic and Applied Biology, School of Sciences, University of Energy and Natural Resources, Sunyani, BA Ghana; 2 Experimental Parasitology Unit, Department of Medical Microbiology, Academic Medical Centre, Amsterdam University Medical Centers, Amsterdam, The Netherlands; 3 Department of Civil Engineering, Kwame Nkrumah University of Science and Technology, Kumasi, AR Ghana; 4 Office of the Vice Chancellor, Catholic University College of Ghana, Sunyani, BA Ghana; 5 Faculty of Health and Allied Sciences, Koforidua Technical University, Koforidua, ER Ghana; 6 Department of Mathematics and Statistics, University of Energy and Natural Resources, Sunyani, BA Ghana

**Keywords:** Onchocerciasis, *Simulium*, Relative abundance, Biting rates, Neglected tropical disease, Ivermectin, Climatic conditions, Modelling

## Abstract

**Background:**

Knowledge of the relative abundance and biting rates of riverine blackflies (vectors of onchocerciasis) is essential as these entomological indices affect transmission of the disease. However, transmission patterns vary from one ecological zone to another and this may be due to differences in species of blackfly vectors and the climatic conditions in the area. This study investigated the effects of climate variability on the relative abundance and biting rates of blackflies in the Tanfiano community (Nkoranza North District, Bono East Region, Ghana). Such information will help to direct policy on effective timing of the annual mass drug administration of ivermectin in the area.

**Methods:**

The study employed human landing collections and locally built Esperanza window traps to collect blackflies from March 2018 to February 2019. The relative abundance and biting rates of the *Simulium* vectors as well as the monthly climatic conditions of the study area were monitored. Correlation analysis and Poisson regression were used to establish the relationships between the variables.

**Results:**

The relative abundance and biting rates of the *Simulium* vectors were highest in the drier months of March, April and August, characterized by high temperatures, low humidity, longer hours of sunshine and stronger winds. The rainy months of May, June and July, characterized by low temperatures, high humidity, few hours of sunshine and weaker winds, had relatively low blackfly abundance and biting activity. Correlation analysis showed that only temperature was significantly, positively correlated with the relative abundance of blackflies (*r* = 0.617, *n* = 12, *P* = 0.033) and monthly biting rates (*r* = 0.612, *n* = 12, *P* = 0.034). A model to predict relative abundance and monthly biting rates using climatological variables was developed.

**Conclusions:**

This study demonstrated that *Simulium* species in the study area preferred higher temperature, lower humidity and rainfall, more hours of sunshine and relatively stronger winds for survival. It is thus recommended that for the study district and others with similar climatological characteristics, mass drug administration of ivermectin should take place in April and September when the abundance of vectors has begun to decline after peaking.
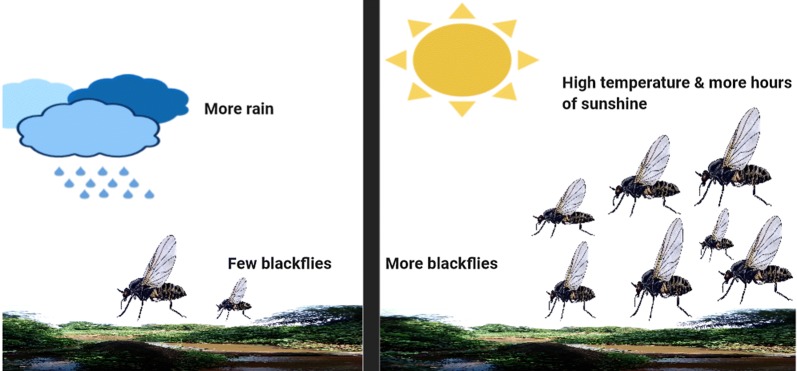

## Background

Onchocerciasis is a neglected tropical disease caused by the nematode *Onchocerca volvulus* and transmitted by riverine *Simulium* (blackfly) vectors [1]. The disease manifests either as ocular or dermal onchocerciasis with several symptoms including itching, nodules, skin thickening, visual impairment and blindness [1]. Onchocerciasis causes great morbidity and is the second leading cause of blindness due to infectious disease globally [2]. Worldwide, it is estimated that there are 37 million people suffering from river blindness and the population at risk is about 120 million [3]. While not deadly, the disease has a global burden of about 1.5 million disability adjusted life years (DALYs) [4]. The severe pruritis (itching) alone accounts for 60% of the DALYs [4]. Meanwhile, these estimates are known to underrepresent the true burden of the disease in endemic regions [5]. Control of onchocerciasis has relied heavily on vector control and mass drug administration using ivermectin, an efficient microfilaricide, which has been effective in interrupting transmission and eliminating the disease in some previously endemic foci [6].

The nature and severity of onchocerciasis in a particular area depends on the type of *Simulium* vector species occurring within a specific ecological zone [7]. In Africa, the *Simulium damnosum* (*sensu lato*) species complex, with about 60 cytoforms, is the main vector responsible for more than 95% of onchocerciasis cases. In West Africa, the vectors are various cytoforms of the *Simulium damnosum* complex, which differ in their ecologies [8] and vectorial roles [9]. Of the principal vectors in West Africa, *S. sanctipauli*, *S. soubrense* and *S. yahense* are found mostly in forests, *S. squamosum* principally occurs in highland zones, while *S. damnosum* (*sensu stricto*) and *S. sirbanum* are more widespread, but the latter two are the only common species found in northern savannah zones [10]. The most important West African sibling species found in Ghana are *S. damnosum* Vajime & Dunbar (*s.s*.), *S. sirbanum* Vajime & Dunbar, *S. sanctipauli* Vajime & Dunbar, *S. yahense* Vajime & Dunbar, *S. soubrense* Beffa form, *S. squamosum* Enderlein (of which both C and E forms occur) and *S. yahense* [11]. The abundant diversity of the species and cytoforms of blackflies in Ghana (and Africa in general) implies that generalizations about effects of climatic variations on onchocerciasis transmission require caution unless the particular vector(s) involved are specified, with similar caveats necessary for different river sizes and bioclimatic zones [12].

Previous studies have demonstrated that transmission of onchocerciasis in an endemic community is related to the relative abundance of the *Simulium* vectors and their biting activities [11, 12]. Meanwhile, the relative abundance and distribution of the vector *S. damnosum* complex (prevalent in Ghana) may be affected by climatic conditions [13]. Studies have reported that climatic variables such as wind speed/direction, temperature, atmospheric pressure, altitude, rainfall, drought, flooding, etc. affect the relative abundance, species habitat and transmission potentials of various diseases especially those transmitted by vectors [10, 13, 14]. The Disease Reference Group for Helminth Infections of the UNICEF/UNDP/World Bank/WHO Special Programme for Research and Training in Tropical Diseases, identified the need to investigate the effects of climate change on helminthiases and their control as well as interactions between the biology of the infections and climate-driven environmental variables [15]. Several studies have investigated the effects of certain environmental variables on the entomological indices of onchocerciasis, but results have been conflicting [13]. However, the general consensus is that different (and sometimes the same) species of *Simulium* in different ecological zones, respond differently to variations in climatic conditions [13]. The need for studies in the different endemic areas to understand the behaviour of the local blackfly vectors is therefore warranted. The aim of this study was to investigate the effects of temperature, rainfall, humidity, hours of sunshine and wind speed on the relative abundance and biting rates of the *Simulium* species in the study community.

## Methods

### Study area

The study took place in Tanfiano in the Nkoranza North District of the Bono East Region of Ghana, West Africa. The community has an ongoing mass drug administration programme for more than a decade (2002–2020), but effective monitoring of the post-control disease dynamics has not been carried out [16]. The community has been shown to be hypoendemic for onchocerciasis [16]. The Nkoranza North district lies within longitudes 1°10″W and 1°55″W and latitudes 7°20″N and 7°55″N [17]. Notable rivers in the district include Tanko, Fanku, Fia and Tanfi. The rivers in the area are fast flowing and provide suitable breeding grounds for the vectors of onchocerciasis [17]. The district lies in the forest-savannah transition zone, hence the predominant blackfly species are *S. damnosum* (*s.s*.), but *S. sirbanum* may also be found in this area [11]. The major rainy season is between April and July and the minor rainy season occurs between September and November [17]. The temperature in the district is generally high with an annual average temperature of 26 °C, with an average maximum temperature of 30.9 °C and average minimum of 21.2 °C. The hottest months are February, March and April [17].

### Vector collection

A longitudinal study was conducted where human landing catches (HLC) and Esperanza window traps were utilized to collect blackflies from March 2018 to February 2019. The period was chosen to cover one major rainy season, one minor rainy season and two dry seasons. This allowed for the evaluation of changes in relative abundance and monthly biting rates of blackflies over different periods with variable climatic conditions. Five different versions of the recently developed Esperanza Window Traps (EWTs) [18] were built and used for the collection of blackflies. The basic design of the trap was made up of a metallic frame with coloured fabrics, CO_2_ sources and host odours source (worn socks) as previously described [19]. Human landing collections were performed along with collections by EWTs. This was because human landing collection is the gold standard for blackfly collection in onchocerciasis programmes, hence it was needed as a control for the relatively new EWTs.

The EWTs and human volunteers were stationed close to the banks of the community river where the blackflies are known to breed. The distance between traps and the river ranged from 2–20 meters following already established protocols [18]. The traps were set at a distance of at least 5 m from each other [18, 20]. These distances have been reported to be adequate to stop interference among trapping methods whilst still permitting the sampling of the same local blackfly populations [18]. Human landing collections were conducted by two well-trained and experienced community volunteers working simultaneously during the period of the trap testing [20]. The fly collectors sat with their legs exposed and any fly perching on the exposed parts were caught before it fed by inverting a small tube over it [20]. Each fly was caught in a different tube and hourly captures were pooled and labelled.

The number of flies caught by the various methods over a period of 12 months were pooled to determine the relative abundance of blackflies. Pooling was necessary due to the low density of blackflies within the community with the resultant low vector catches from all trapping methods over the 12 months period. The pooled data was also used to determine the monthly biting rates (MBR) following previously published procedures [14] using the formula:

$$ MBR \, = {{(Number \, of \, flies \, caught \times Days \, in \, the \, month)} \mathord{\left/ {\vphantom {{(Number \, of \, flies \, caught \times Days \, in \, the \, month)} {Number \, of \, catching \, days}}} \right. \kern-0pt} {Number \, of \, catching \, days}} $$

### Climatological data

The monthly average rainfall, humidity, temperature, sunshine hours and wind speed were acquired from the international water management institute [21]. The GPS coordinates of Tanfiano are 7°46′60″N, 1°46′0″W [22] and this was used to generate the climatic data for the months of March 2018 through to February 2019 from the institute.

### Statistical analysis

The data gathered were recorded in Microsoft Excel and analyzed in GraphPad Prism (version 8; GraphPad Software, San Diego, CA, USA) and R Package for Statistical Computing (version 3.6.1) [23]. The Shapiro-Wilk test was used to determine if the variables were normally distributed (normal distribution was assumed if *P* > 0.05). The Pearson correlation test was then used to determine the association between the monthly biting rates, the relative abundance and the individual climate variables. The correlation was considered statistically significant at the 95% confidence interval if *P* < 0.05. The dependent variable (relative abundance of blackflies) was count data, hence Poisson regression was employed to model the relationship between relative abundance, monthly biting rates and the climatological variables.

## Results

**Fig. 1:**
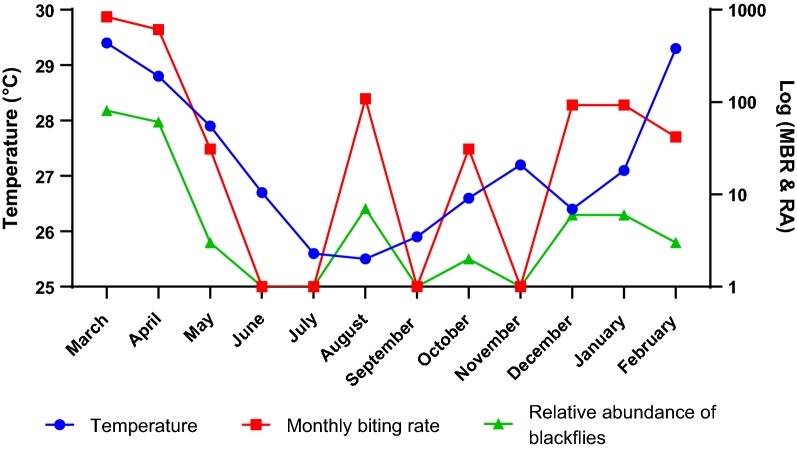
Relationship between mean monthly temperature (in °C), monthly biting rate (MBR) and relative abundance (RA) of blackflies in Tanfiano from March 2018 to February 2019

The correlation between selected climatic variables and the relative abundance and the MBR of the *Simulium* vectors over the study period are presented in the figures below. The climatic conditions observed during the study period were typical for the study area [17]. To calculate the MBR and relative abundance for this study, the blackflies captured by the EWTs and HLCs were pooled together. The highest number of captured blackflies (*n* = 81) was recorded in March followed by April (*n* = 61) (Fig. 1). In May, the number of blackflies reduced drastically as only 3 blackflies were caught by EWTs and HLCs combined. The months of June and July recorded no catches and the blackflies seemed to disappear completely. In August, the *Simulium* vectors started re-appearing and 7 were caught by EWTs and HLCs combined. However, in September and November no blackflies were caught, and in October just 2 were caught. Some blackflies were caught in December (*n* = 6), January (*n* = 6) and February (*n* = 3).

The study investigated the effects of variation in temperature on the MBR and the relative abundance of *Simulium* vectors (Fig. 1). The mean monthly temperature was highest in March (29.4 °C) reducing steadily until July (25.6 °C). The mean temperature of August (25.5 °C) was only slightly lower than that of July (25.6 °C). From September to November the mean temperature steadily increased until December when it dropped to 26.4 °C. Generally, both the relative abundance and the MBR of blackflies decreased with decreasing temperature (Fig. 1). The MBR and relative abundance of the blackflies were highest in March (837 and 81 flies/man/month, respectively) when the temperature was highest (29.4 °C).

The mean monthly temperature was normally distributed (Shapiro-Wilk test, *W* = 0.915, *P* = 0.246) hence the Pearson correlation test was used to determine the relationship between temperature and relative abundance of the blackflies. The correlation was moderately positive and statistically significant (Pearsonʼs correlation coefficient, *r* = 0.617, *n* =12, *P* = 0.033). There was also a moderately positive correlation between the temperature and the MBR and this correlation was statistically significant (*r* = 0.612, *n* = 12, *P* = 0.034).

**Fig. 2:**
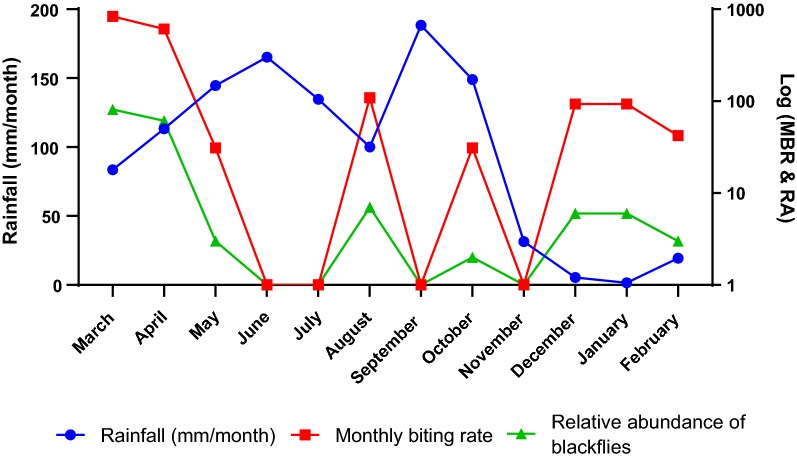
Relationship between mean rainfall (in mm/month) and monthly biting rate (MBR) and relative abundance (RA) of *Simulium* vectors in Tanfiano from March 2018 to February 2019

The study also investigated the mean monthly rainfall and its relationship with the MBR and relative abundance of the blackflies (Fig. 2). The analysis showed that as rainfall increased the relative abundance and MBR also decreased. For instance, the MBR and relative abundance of the blackflies were highest in March (837 and 81 flies/man/month, respectively) when the rainfall was low (83.49 mm/month). As the rainfall increased to 113.19 mm/month, the MBR and relative abundance decreased to 610 and 61 flies/man/month, respectively. In May, as the rainfall further increased to 144.67 mm/month, the MBR and relative abundance declined further to 31 and 3 flies/man/month, respectively. In June, as the rain peaked at 165.16 mm/month, both MBR and relative abundance of the blackflies declined to zero. It is worth noting that as the rains reduced in July, the reappearance of the blackflies was not immediate, and it was not until August that they started to reappear.

The mean monthly rainfall was normally distributed (Shapiro-Wilk test, *W* = 0.918, *P* = 0.260) hence the Pearson correlation test was used to determine the relationship between rainfall and relative abundance of the blackflies. The correlation was weakly negative and not statistically significant (*r* = − 0.041, *n* = 12, *P* = 0.899). Meanwhile, there was also a weak negative correlation between the mean monthly rainfall and the MBR of the *Simulium* vector and this correlation was not statistically significant (*r* = -0.073, *n* = 12, *P* = 0.822).

**Fig. 3:**
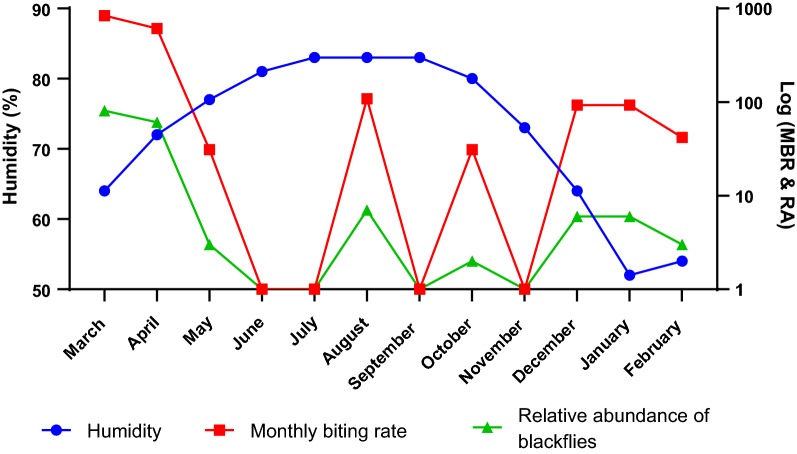
Relationship between relative humidity (in %), monthly biting rate (MBR) and relative abundance (RA) of blackflies in Tanfiano from March 2018 to February 2019

The relationship between the relative humidity, MBR and the relative abundance of *Simulium* vectors is presented in Fig. 3. The humidity of the study area continuously increased from low (64%) at the onset of the study in March to very high (83%) at the end of August. Generally, as humidity increased, the MBR and the relative abundance decreased until the month of June and July, when in spite of the increasing humidity, there was no appreciable change in the MBR and relative abundance.

The relative humidity was normally distributed (Shapiro-Wilk Test, *W* = 0.866, *P* = 0.057) hence the Pearson correlation test was used to determine the relationship between humidity and relative abundance of the blackflies. The correlation was mildly negative and not statistically significant (*r* = − 0.237, *n* = 12, *P* = 0.459). Furthermore, there was a mild, negative correlation between the humidity and the MBR but this was also not statitstically significant (*r* = − 0.260, *n* = 12, *P* = 0.415*)*.

**Fig. 4:**
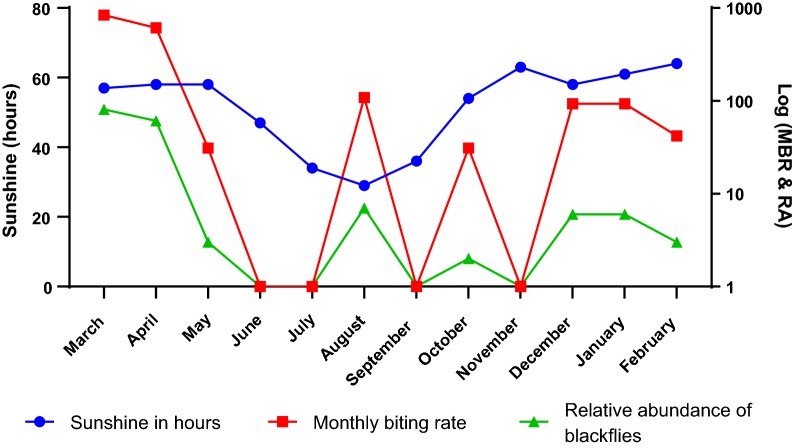
Relationship between sunshine (% of hours), monthly biting rate (MBR) and relative abundance (RA) of blackflies in Tanfiano from March 2018 to February 2019. Monthly % sunshine hours represent the percentage of hours in a month with sunshine

The effect of hours of sunshine in the month on the MBR and the relative abundance of the blackflies was also evaluated (Fig. 4). The hours of sunshine for the study duration slightly increased from March until May. Between June and July, there was a decline in sunshine hours as the rains were heavy until September when it began to increase again.

The distribution of the monthly sunshine hours was normalized by log-transformation (Shapiro-Wilk test, *W* = 0.839, *P* = 0.057) and the Pearson correlation test was used to determine the relationship between sunshine hours and relative abundance of the blackflies. The correlations were mildly positive for both the relative abundance (*r* = 0.230, *n* = 12, *P* = 0.472) and the MBR (*r* = 0.227, *n* = 12, *P* = 0.478) but the relationships were not statistically significant.

**Fig. 5:**
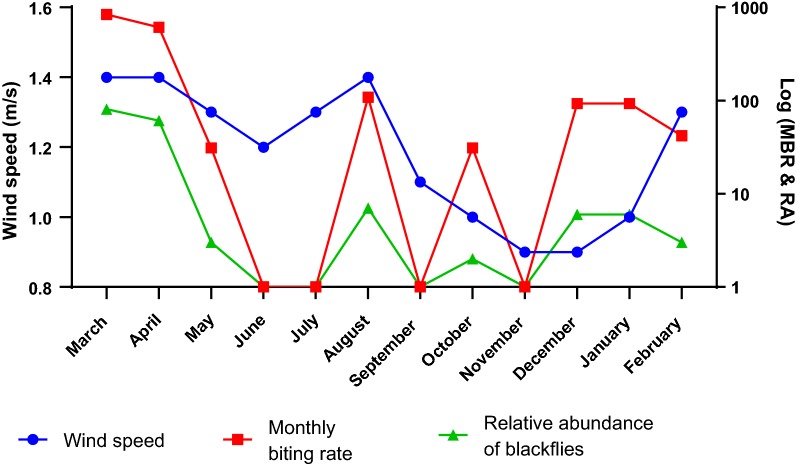
Relationship between monthly average wind speed, monthly biting rate (MBR) and relative abundance (RA) of blackflies in Tanfiano from March 2018 to February 2019

The relationship between the monthly average wind speed (m/s), the MBR and the relative abundance was also interesting (Fig. 5). In March, when the average wind speed was very high (1.4 m/s), both the MBR and relative abundance were also correspondently very high (837 and 81 flies/man/month, respectively). The months of April and June, with decreasing wind speed, had reductions in the relative abundance and the MBR of the blackflies. The general trend demonstrated in this study is that with decreasing wind speed, the relative abundance and the MBR also decreased.

The wind speed was normally distributed (Shapiro-Wilk test, *W* = 0.873, *P* = 0.071) hence the Pearson correlation test was used to determine the relationship between wind speed and relative abundance of the blackflies. Analysis of the correlation showed that wind speed was moderately, positively correlated with both the relative abundance (*r* = 0.515, *n* = 12, *P* = 0.087) and the MBR (*r* = 0.506, *n* = 12, *P* = 0.093) of the vectors as shown in Fig. 5. However, both relationships were not statistically significant.

The study further modelled the relationship between the MBR, relative abundance, temperature, rainfall, humidity, sunshine and wind speed. The results showed that MBR can accurately be predicted using a model that incorporates relative abundance and all climate variables except for humidity. Humidity was excluded from the final model because it was not a significant predictor (*P* = 0.596). The results of the analysis are presented in Table 1.

**Table 1:** Poisson model of the relationship between MBR, RA and climate variables

Variable	Estimate	SE	*Z*-value	*P*-value
Intercept	12.419122	1.291834	9.614	< 0.0001
Relative abundance	0.054997	0.002275	24.16	< 0.0001
Temperature	− 1.928488	0.134700	− 14.317	< 0.0001
Rainfall	− 0.017507	0.001488	− 11.762	< 0.0001
Sunshine	18.570692	1.343811	13.819	< 0.0001
Wind speed	11.008602	0.810060	13.590	< 0.0001

*Notes*: Null deviance: 3946.33 on 11 degrees of freedom, residual deviance: 176.93 on 6, AIC: 241.16

*Abbreviation*: SE, standard error

In the final model, all coefficients of the model were highly significant even at 99% confidence interval (Table 1). This final model explained 95.52% variability in the monthly biting rate (MBR) of blackflies in the community. This means that the final fit can predict with 95.52% accuracy. The mathematical representation of the final model is given in equation 1 as:

1 $$ \begin{aligned} Log_{e} MBR =\, & { 12}. 4 1 9 1 2 2+ 0.0 5 4 9 9 7\times \, RA \, - 1. 9 2 8 4 8 8\times \, Temperature \, - 0.0 1 7 50 7\\ & \times \, Rainfall + 1 8. 5 70 6 9 2\times \, Sunshine + 1 1.00 8 60 2\times \, Wind \, speed \\ \end{aligned} $$

**Fig. 6:**
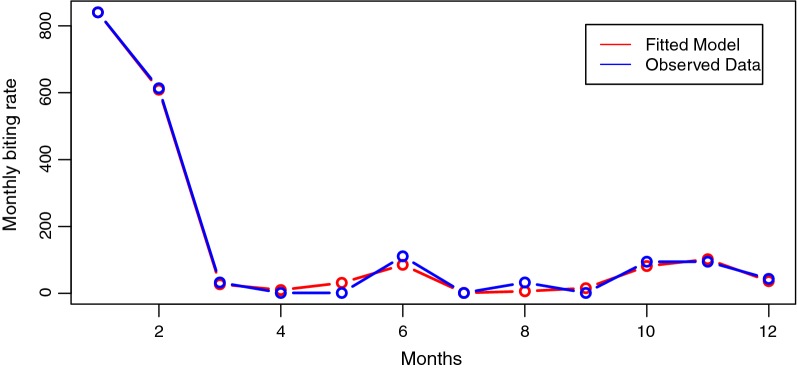
Plot of observed MBR data over fitted model at 95% confidence interval

To check for the accuracy of the model, a plot of the observed data over the fitted model was made and is presented in Fig. 6. The plot demonstrated that the model fits the data accurately as there were only a few observed variations between the observed data points and the fitted model.

The study also developed a model to predict the relative abundance of blackflies using only the climatological variables and the results of the model are presented in Table 2. Due to the fact that wind speed was not a significant predictor (*P* = 0.3326), it was dropped in the final model fitted for the relative abundance.

**Table 2:** Poisson model of the relationship between RA and climate variables

Variable	Estimate	SE	*Z*-value	*P*-value
Intercept	− 47.326379	6.762367	− 6.998	< 0.0001
Temperature	2.026700	0.200949	10.086	< 0.0001
Sunshine	− 9.374448	1.837439	− 5.102	< 0.0001
Humidity	0.150918	0.038089	3.962	< 0.0001
Rainfall	− 0.013825	0.005554	− 2.489	< 0.0128

*Notes*: Null deviance: 405.621 on 11 degrees of freedom, residual deviance: 104.08 on 7 degrees of freedom, AIC: 45.99

*Abbreviation*: SE, standard error

In the final model, all the coefficients of the climate variables were highly significant at 95% confidence level. This new fit explained 74.34% of the variability in the relative abundance of blackflies in the community. The model is given in Equation 2 as:

2 $$ \begin{aligned} Loge RA =\, &  0.150918 \times Humidity - 47.326379 + 2.026700 \times Temperature  \\ & - 0.013825 \times Rainfall - 9.374448 \times Sunshine  \\ \end{aligned} $$

**Fig. 7:**
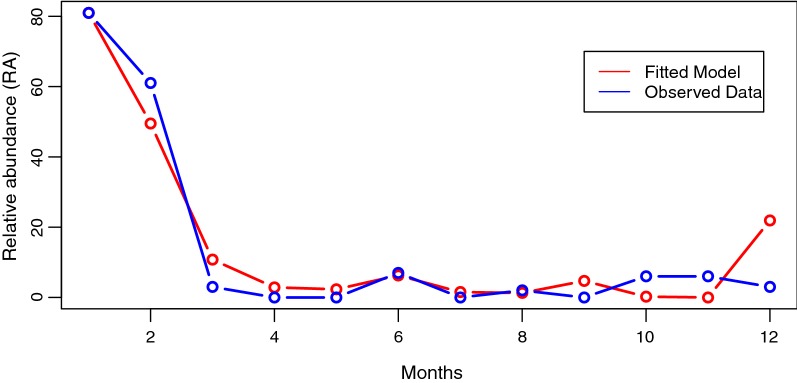
Plot of observed RA data over fitted model at 95% confidence interval

In order to test the accuracy of the final model, a plot of the observed data over the fitted model was made (Fig. 7). The results demonstrated that the model provided a good fit for the data.

## Discussion

The study observed that the monthly biting rates and relative abundance of the *Simulium* vectors were highest in the drier months of March, April and August in the study area (Figs 1, 2, 3, 4 and 5). The drier months were conspicuously marked by higher temperature, lower rainfall, lower humidity, more hours of sunshine and stronger winds. The findings in this study are consistent with that of Eyo et al. [24] who demonstrated that high temperature and low humidity correlated with a high relative abundance of *S. damnosum*. Additionally, Ikpeama et al. [25] reported that blackflies preferred higher temperatures, as is the case with other hematophagous insects. Nevertheless, extremely high temperatures have also been shown to negatively affect the biting rates of certain *Simulium* vectors [26].

The rainy months of May to July had relatively lower temperatures with fewer hours of sunshine, high humidity and weaker winds. For these months, the study observed relatively low fly abundance and biting activity. This could be an advantage for a predominantly farming community such as in the study area, where farming activities intensify in the rainy seasons. This means that inhabitants may be at a reduced risk of being exposed to bites of the *Simulium* vectors when they go to work on the farm. This might also explain why there was a relatively low prevalence of onchocerciasis in the study area as reported by Otabil et al. [16]. The findings of the present study are similar to that of Zarroug et al. [14] carried out in Sudan and Ubachukwu & Anya [26] carried out in Nigeria, and demonstrated that a surge in the amount of rainfall correlated with a decrease in the relative abundance of the *Simulium* vectors. The reason for the seeming disappearance of the *Simulium* vectors may be due to heavy downpours, as it is possible that rivers could overflow their banks and sweep away larvae and adult flies [24]. Indeed, a study by Nwoke [27] showed that rainfall increases relative humidity leading to lower temperatures and this has the potential to negatively affect the blackfly biting activity [27]. The low temperatures during rainy periods might also impede the development of the *Simulium* larvae. However, results from this study are divergent to findings by Atting et al. [28] and Opara et al. [29] in Nigeria reporting that the number of *S. damnosum* increased during periods when rainfall was at its peak. Opara et al. [29] suggested that the increase in fly abundance may be due to the increase in oxygen in the breeding habitats, rivers and streams [29]. Likewise, an increase in the oxygen content of breeding sites may be attended by an increase in the amount of nutrients and availability of pre-imaginal sites [24]. This could lead to an enhanced pre-imaginal growth, which could result in a rise of the adult blackfly population [24] during the rainy season (May to July).

However, for the present study, the rainy months had lower temperatures, higher humidity, fewer hours of sunshine and relatively weaker winds. The climatic conditions observed in the study period were typical for the study area and its surroundings [17]. All the climatic conditions seemed to have interplayed to bring about the observed reduction in fly abundance and biting rates. The disparity in ecological and climatic requirements of different species of *Simulium* is a widely known fact, and the published data indicates that it is always essential to conduct area-specific entomological studies in the different endemic communities [13] in order to know what pertains in the particular area.

One of the key implications of the findings of this study is that onchocerciasis control programmes must carry out epidemiological surveys in control areas before scheduling mass drug administration of ivermectin. This is because the biting rates and relative abundance of blackflies are very high in some periods of the year and low in others. It will therefore be best to consider administering ivermectin during periods when these entomological indices are high as individuals would have been highly exposed to blackfly bites. For the present study community, our results indicate that it would be best to administer ivermectin in April and September immediately following the peak periods of exposure to blackflies.

Our study further developed models to predict the MBR and RA using climate variables. The findings from the study showed that for the study area, the models (Equations 1 and 2) can accurately predict these key indices of transmission of onchocerciasis if the climatological variables are known. The need for such models has been underscored by previous authors [13, 15]. However, there is a need for external validation of the models developed in this study in order to increase their utility in current onchocerciasis control programmes in the area.

## Conclusions

The present study established that entomological indices of onchocerciasis are affected by the climatic conditions in an area. It has also shown that temperature, rainfall, wind, sunshine and humidity have varied effects on the *S. damnosum* vectors in the study area and their vectorial capacity. Juxtaposing the findings of this research to several others in other endemic areas, it is evident that different ecological forms of the *Simulium* vectors react differently to climatic variability. In light of concerns about the rapidly changing global climate, there is a need to constantly monitor entomological indices of onchocerciasis in order to ensure that onchocerciasis elimination targets are not adversely affected by the changing climate. This study demonstrated that *Simulium* species in the study area preferred higher temperature, lower humidity and rainfall, more hours of sunshine and relatively strong winds for survival. It is thus recommended that for the study district and others with similar characteristics, mass drug administration of ivermectin should be administered in April and September (biannual administration), when the number of vectors have begun to decline after the peak of exposure to blackflies.

## Data Availability

Data supporting the conclusions of this article are included within the article. The datasets used and/or analyzed during the present study are available from the corresponding author upon reasonable request.

## Abbreviations

CO_2_: carbon dioxide
DALYs: disability adjusted life years
EWTs: Esperanza window traps
GPS: global positioning system
HLC: human landing catches
MDA: mass drug administration
MBR: monthly biting rate
UNDP: United Nations Development Programme
UNICEF: United Nations International Childrenʼs Emergency Fund
WHO: World Health Organization

## References

[1] WHO. Onchocerciasis elimination mapping of endemic countries is key to defeating river blindness, Geneva: World Health Organisation; 2019. https://www.who.int/neglected_diseases/news/Onchocerciasis-elimination-mapping-of-endemic-countries-is-key/en/. Accessed 11 Jul 2019.

[2] Turner HC, Walker M, Lustigman S, Taylor DW, Basánez M (2015). Human onchocerciasis: modelling the potential long-term consequences of a vaccination programme. PLoS Negl Trop Dis..

[3] WHO/Ministry of Health, Ghana. The expanded special project for elimination of neglected tropical diseases. 2019. http://espen.afro.who.int/countries/ghana. Accessed 14 Apr 2019.

[4] Remme JHF, Feenstra P, Lever PR, Medici AC, Morel CM, Noma M, et al. Tropical diseases targeted for elimination: chagas disease, lymphatic filariasis, onchocerciasis, and leprosy. In: Jamison DT, Breman JG, Measham AR, et al., editors. Disease Control Priorities in Developing Countries. 2nd edition. Washington (DC): The International Bank for Reconstruction and Development/The World Bank; 2006. Chapter 22. https://www.ncbi.nlm.nih.gov/books/NBK11745/ Co-published by Oxford University Press, New York. 21250324

[5] Basáñezz MG, Pion SDS, Churcher TS, Breitling LP, Little MP, Boussinesq M (2006). River blindness: a success story under threat?. PLoS Med..

[6] Wanji S, Kengne-Ouafo JA, Esum ME, Chounna PWN, Tendongfor N, Adzemye BF (2015). Situation analysis of parasitological and entomological indices of onchocerciasis transmission in three drainage basins of the rain forest of South West Cameroon after a decade of ivermectin treatment. Parasit Vectors..

[7] Umeh RE, Mahmoud AO, Hagan M, Wilson M, Okoye OI, Asana U (2010). Prevalence and distribution of ocular onchocerciasis in three ecological zones in Nigeria. Afr J Med Sci..

[8] Vajime CG, Dunbar RW (1975). Chromosomal identification of eight species of the subgenus *Edwardsellum* near and including *Simulium* (*Edwardsellum*) *damnosum* Theobald (Deptera: Simuliidae). Tropenmed Parasitol..

[9] Cheke RA, Garms R (2013). Indices of onchocerciasis transmission by different members of the *Simulium damnosum* complex conflict with the paradigm of forest and savanna parasite strains. Acta Trop..

[10] Post RJ, Cheke RA, Boakye DA, Wilson MD, Osei-Atweneboana MY, Tetteh-Kumah A (2013). Stability and change in the distribution of cytospecies of the *Simulium damnosum* complex (Diptera: Simuliidae) in southern Ghana from 1971 to 2011. Parasit Vectors..

[11] Lamberton PHL, Cheke RA, Walker M, Winskill P, Osei-Atweneboana MY, Tirados I (2014). Onchocerciasis transmission in Ghana: biting and parous rates of host-seeking sibling species of the *Simulium damnosum* complex. Parasit Vectors..

[12] Lamberton PHL, Cheke RA, Winskill P, Tirados I, Walker M, Osei-Atweneboana MY (2015). Onchocerciasis transmission in Ghana: persistence under different control strategies and the role of the simuliid vectors. PLoS Negl Trop Dis..

[13] Cheke RA, Basáñez MG, Perry M, White MT, Garms R, Obuobie E (2015). Potential effects of warmer worms and vectors on onchocerciasis transmission in West Africa. Philos Trans R Soc B Biol Sci..

[14] Zarroug IMA, Hashim K, Elaagip AH, Samy AM, Frah EA, ElMubarak WA (2016). Seasonal variation in biting rates of *Simulium damnosum sensu lato*, vector of *Onchocerca volvulus*, in two sudanese foci. PLoS ONE..

[15] Basáñez M-G, McCarthy JS, French MD, Yang G-J, Walker M, Gambhir M (2012). A research agenda for helminth diseases of humans: modelling for control and elimination. PLoS Negl Trop Dis..

[16] Otabil KB, Gyasi SF, Awuah E, Obeng-Ofori D, Atta-Nyarko RJ, Andoh D (2019). Prevalence of onchocerciasis and associated clinical manifestations in selected hypoendemic communities in Ghana following long-term administration of ivermectin. BMC Infect Dis..

[17] Ghana Statistical Service. Ghana Statical Service 2010 population and housing census docuemnt. http://www2.statsghana.gov.gh/docfiles/2010_District_Report/Brong%20Ahafo/NKORANZA%20North.pdf. Accessed 27 Jan 2019.

[18] Hendy A, Sluydts V, Tushar T, Witte JD, Odonga P, Loum D (2017). Esperanza window traps for the collection of anthropophilic blackflies (Diptera: Simuliidae) in Uganda and Tanzania. PLoS Negl Trop Dis..

[19] Rodríguez-Pérez MA, Adeleke MA, Burkett-Cadena ND, Garza Hernández JA, Reyes-Villanueva F, Cupp EW (2013). Development of a novel trap for the collection of black flies of the *Simulium ochraceum* complex. PLoS One..

[20] Toé LD, Koala L, Burkett-Cadena ND, Traoré BM, Sanfo M, Kambiré SR (2014). Optimization of the Esperanza window trap for the collection of the African onchocerciasis vector *Simulium damnosum sensu lato*. Acta Trop..

[21] International Water Management Institute. IWMI Online Climate Service Model; 2019. http://wcatlas.iwmi.org/. Accessed 11 Jul 2019.

[22] Getmap. Tanfiano - Brong-Ahafo Map, Weather and Photos—Ghana: populated place—Lat:7.78333 and Long:-1.76667. 2019. http://www.getamap.net/maps/ghana/ghana_(general)/_tanfiano/. Accessed 11 Jul 2019.

[23] R Core Team (2013). R: A language and environment for statistical computing. R Foundation for Statistical Computing, Vienna, Austria. http://www.R-project.org/.

[24] Eyo JE, Ikechukwu EO, Ubachukwu PO, Ivoke N, Ekeh FN (2014). Effects of climatic conditions on the biting density and relative abundance of *Simulium damnosum* complex in a rural Nigerian farm settlement. Ann Agric Environ Med..

[25] Ikpeama CA, Nwoke BEB, Anosike JC (2006). Studies on the ecology and ditribution of blackflies (Diptera: Simuliidae) in Imo state Nigeria. Int J Nat Appl Sci..

[26] Ubachukwu P, Anya A (2006). Studies on the diurnal biting activity pattern of *Simulium damnosum* complex (Diptera: Simuliidae) in Uzo-Uwani local government area of Enugu state, Nigeria. Niger J Parasitol..

[27] Nwoke BEB (1988). Studies on the field epidemiology of human onchocerciasis on the Jos Plateau, Nigeria-VII. The effects of climatic factors on the diurnal biting behaviour of *Simulium damnosum* Theobald (Diptera: Simuliidae). Int J Trop Insect Sci..

[28] Atting IA, Ejezie GC, Braide EI, Opara KN, Ekwe A (2008). Seasonal variations in human onchocerciasis transmission by black flies *Simulium damnosum* in a forest area of Cross River State, Nigeria. Afr J Appl Zool Environ Biol..

[29] Opara KN, Usip LP, Akpabio EE (2008). Transmission dynamics of *Simulium damnosum* in rural communities of Akwa Ibom State, Nigeria. J Vector Borne Dis..

